# Description and validation of a circular padding method for linear roughness measurements of short data lengths

**DOI:** 10.1016/j.mex.2020.101122

**Published:** 2020-11-17

**Authors:** Stijn Schoeters, Wim Dewulf, Jean-Pierre Kruth, Han Haitjema, Bart Boeckmans

**Affiliations:** a MaPS, Department of Mechanical Engineering, KU Leuven, Celestijnenlaan 300, 3001 Heverlee, Belgium; b Member of Flanders Make, Gaston Geenslaan 8, 3001 Heverlee, Belgium

**Keywords:** Surface topography, Profilometry, Roughness, Waviness, Data Filtration, Dimensional Metrology, AM, Additive manufacturing, PBF, Powder bed fusion, SLM, Selective laser melting, NP, Non-padded, CP, Circularly padded, GR, Gaussian regression

## Abstract

Surface topography measurements are vital in industrial quality control. Linear roughness measurements are among the most preferred methods, being quick to perform and easy to interpret. The ISO 16610 standard series prescribes filters that can be used for most cases, but has limitations for restricted measurement lengths. This is because the standard filter type is a Gaussian filter, which like most instances of kernel convolution filters has no output near the edges of the profile, effectively shortening the length of the filtered output profile as compared to the input. In some cases, this leads to a lack of representative data after filtration. Especially in fields such as Additive Manufacturing (AM) this becomes a problem, due to the high “roughness to measurable data length”-ratio that characterizes complex AM parts. This paper describes a method that allows to overcome this limitation:

• A method for circular padding of short measured tracks is described and validated.
• A flexible profile data post-processing tool was developed in MATLAB to grant users more control over the data analysis.

Results obtained from roughness profiles long enough for normal ISO procedures are shown to not change significantly when circularly padded. When only a shorter section of the data is available, where the standard protocol would not be able to compute a filtered profile and related parameters anymore, the circular padding method is shown to lead to results that are in good agreement with the ISO standard procedures.

## Graphical abstract

**Figure:**
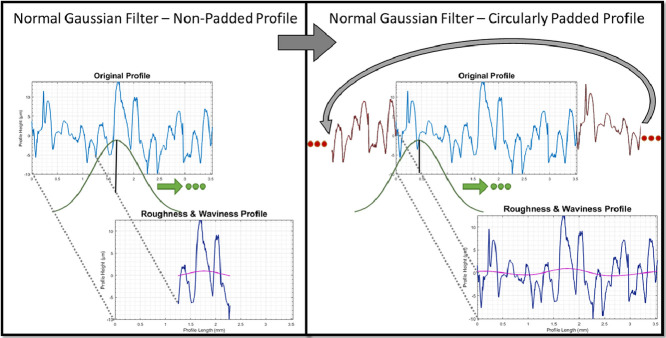

**Table:** Specifications Table

Subject Area:	Engineering
More specific subject area:	*Dimensional Metrology - Surface Profilometry*
Method name:	*Circular Padding*
Name and reference of original method:	*ISO 16610-28:2016: Geometrical product specifications (GPS) - Filtration - Part 28: Profile filters: End effects*
Resource availability:	/

## Method details

*Introduction and problem statement*

Whenever an object gets its functionality from its surfaces, such as a honed engine piston borehole, the quality of those surfaces needs to be assessed. The most common method of analyzing a surface is through the use of a tactile stylus profilometer, resulting in a linear surface profile. Industrially, due to time restrictions for quality control, this fast linear type of measurement with a normal Gaussian convolution filter is still the preferred approach [1,2]-(annex C), which is why this method is the focus of this work.

**Fig. 1:**
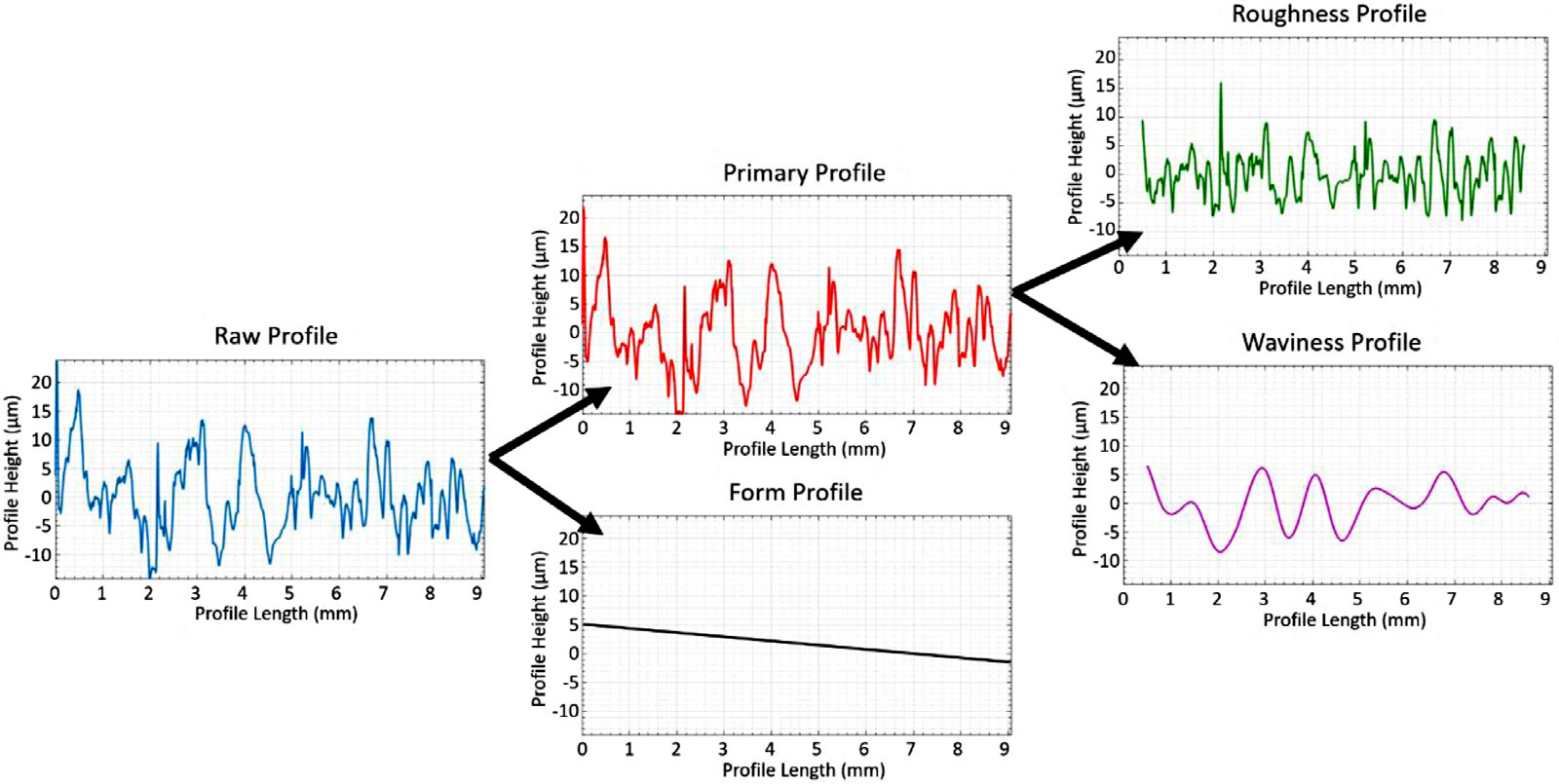
Profile segmentation into the different subtypes of surface textures (after **[****3****]**).

**Fig. 2:**
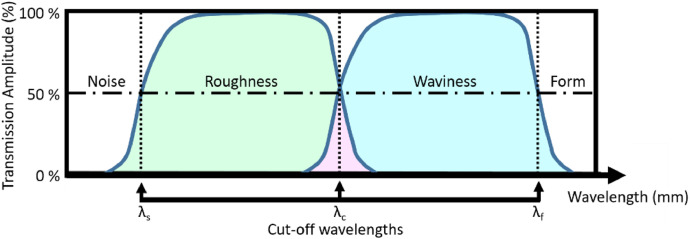
Transmission of amplitudes of wavelengths in the profile using Gaussian filters to segment into roughness and waviness (after [3]).

The measured "raw" profile can be split into different parts: form, waviness and roughness, as shown in Fig. 1 [3]. The decomposition is usually made as described by ISO 4287:1997 [4] and is therefore based on Gaussian filtration. This filtration uses 3 cut-off wavelengths: *λ_s_, λ_c_* and *λ*_f_ as described in ISO 16610:21(2011) [5], dividing the profile into noise, roughness, waviness and form components. A sketch of the transmission amplitudes of wavelengths of the profile is shown in Fig. 2.

The assessment of a measured profile is performed as follows: a first filtration is done using *λ_s_* as the cutoff wavelength, as a low-pass filter, to filter high-frequency noise and probe size effects. Secondly, the form can be filtered further using a high-pass filter with *λ_f_*. Next, the separation of the remaining "primary"-profile is done using a filtration with *λ_c_*, where the higher wavelengths make up the waviness profile and the shorter wavelengths the roughness profile. Finally, profile parameters can then be calculated for any of these profiles. For form, it is common to use a-priori design knowledge instead of a wavelength based filtration. Arc sections, lines and even complete "nominal surface"-profiles can be used to separate this form and the primary profile from the raw data.

The normal Gaussian filtration works by convoluting a pre-determined kernel, a weighting function, with the primary profile. This results in the waviness profile and roughness profile, the latter being the difference between the primary and waviness profiles. This kernel is shaped according to ISO 16610–21:2011 [5] and has infinite support, but approaches zero quite quickly away from the center.

**Fig. 3:**
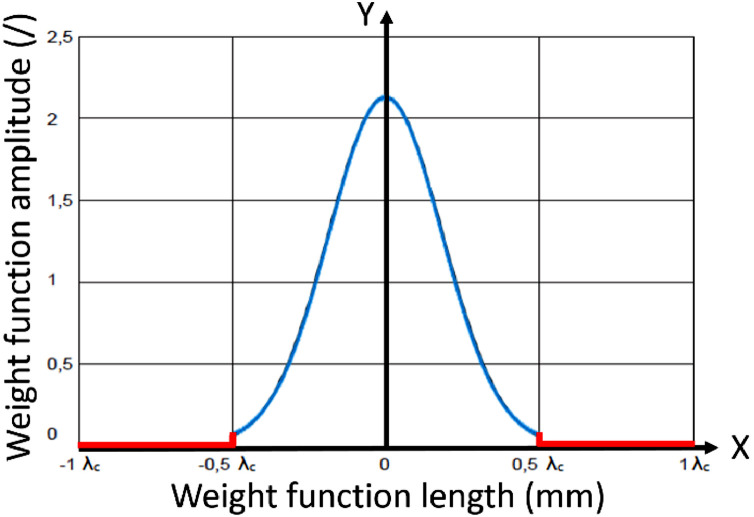
Truncated Gaussian Weighting function – In this figure truncation is set at ½ *λ_c_*, beyond which the value is set to 0. For the lowest error this truncation should be chosen to be *λ_c_* according to ISO 16610–21:2011 (after [5]).

For such a non-truncated kernel function, the convolution would need an infinite length of input data on either side of the center of the weighting function, where the output value is calculated. As is shown in that standard, as well as in Fig. 3, this kernel is therefore normally truncated on either side, so it can be used in convolution with finite data lengths. The ISO standard suggests this truncation to be *λ_c_* at both sides, instead of the depicted *λ_c_*/2 in Fig. 3, for minimal errors in the end result. By truncating this weighting function to a specific width it can be used for convolution with finite length input data, given that it is long enough (i.e. at least 2·*λ_c_*). The standard describes that truncation can be chosen to be ½ *λ_c_*, as shown in Fig. 3, but that for minimal implementation error it should in fact be truncated at *λ_c_* on either side.

**Fig. 4:**
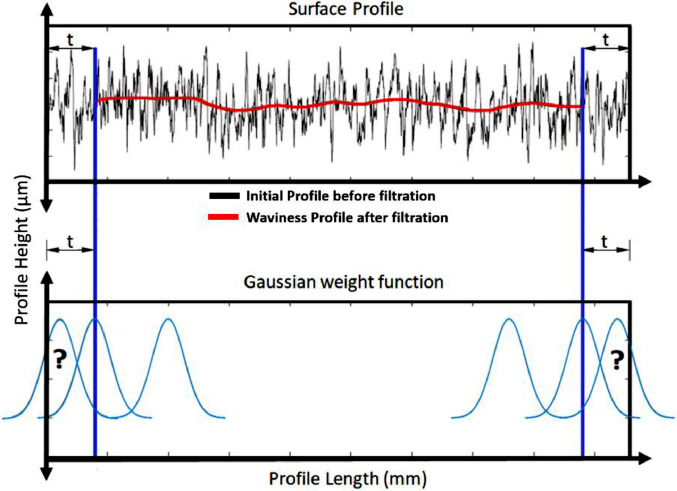
End effects using Gaussian convolution filter (after [6]).

The end result of this convolution is therefore that the resulting profile after convolution filtering has a shorter length than the initial profile. The output can only be calculated starting from a distance equal to this truncation width “t” away from the edges of the profile, shown in red in Fig. 4. Important to note is that all data is indeed used to calculate the output values, but that the output length is shorter, and therefore profile parameters are calculated over a shorter length. This is referred to as “end effects”: see ISO 16610-28:2016 - Geometrical product specifications (GPS) - Filtration - Part 28: Profile filters: End effects [6].

A measurement length of 8 · *λ_c_* is often suggested, as this leads to a filtered output profile length more than the 5 · *λ_c_* minimum that ISO recommends for the calculation of the different profile parameters among which *R_z_* that requires analyzing the filtered profile over 5 times *λ_c_*. In fact, ISO 4288:1996 [7] officially recommends at least 5 · *λ_c_* as the measurement length, before filtration. On the other hand, some manufacturers of profilometers mention a measurement length of 6 · *λ_c_* resulting in a 5 · *λ_c_* evaluation length. This indicates that their post-processing software likely uses a truncation value of ½ *λ_c_* [8].

Objects that are shorter than these limits need to be analyzed differently, as the output profile length is significantly shortened by the convolution "end effects". This is a situation that arises quite often when assessing complex or freeform shapes produced by e.g. Additive Manufacturing. ISO 16610:28 prescribes a few ways to tackle this [6], however in this paper circular padding, as a possible way to deal with this issue efficiently, is proposed and discussed. In this work circular padding will be described in more detail, validation tests will be discussed and best practice guidelines will be established.

## State of the art and proposed method for short measurement tracks

### Zero padding and mirror padding before filtration

**Fig. 5:**
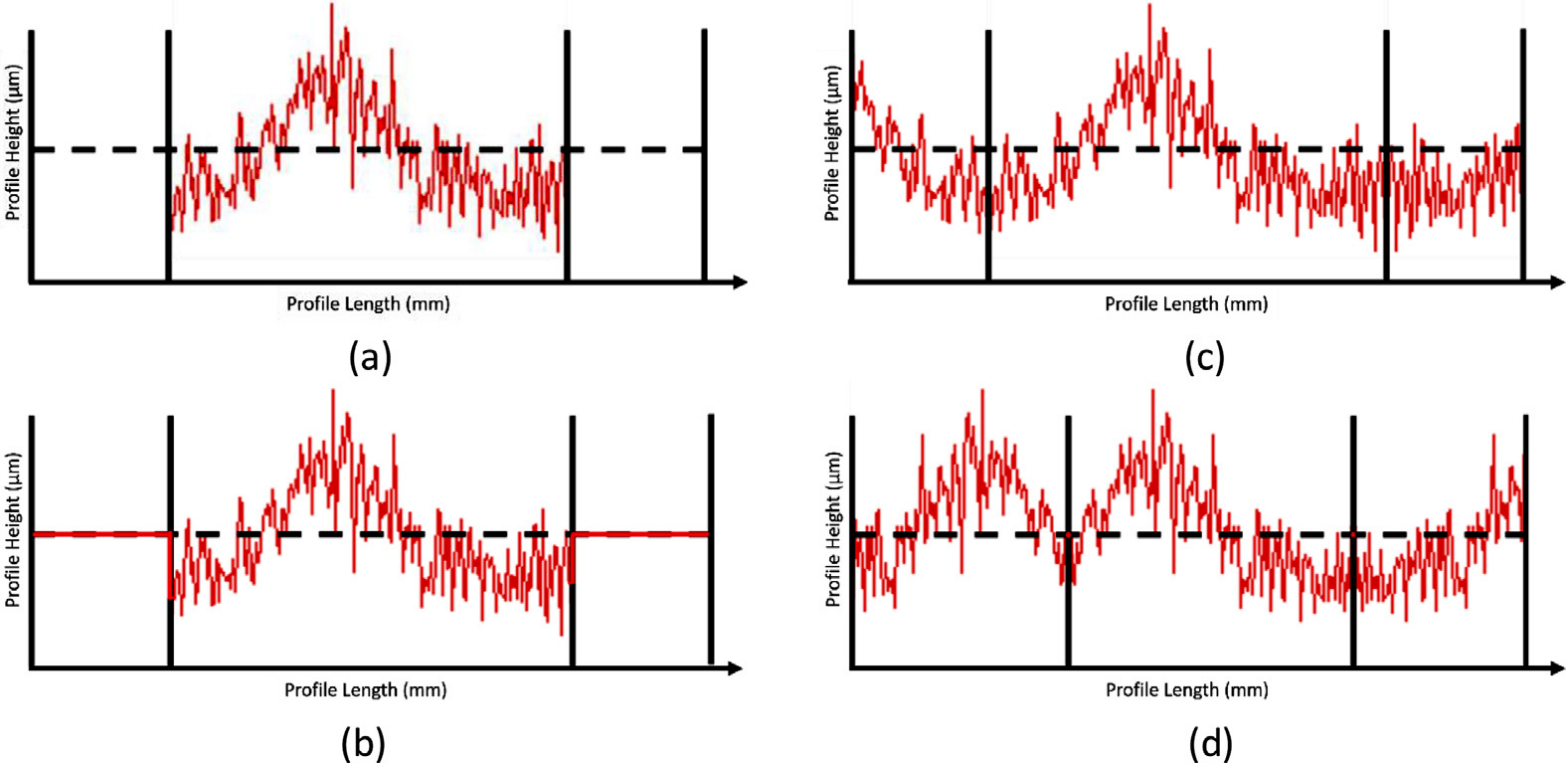
Starting profile (a), zero-padded profile (b) and mirror-padded profiles on different mirroring points (c and d).

The problem of too short a measuring length can be addressed by padding, i.e. by adding virtual data before and after the actual measured profile (Fig. 5). ISO 16610 proposes, among others, “zero-padding”: adding zeros before and after the measured data. See the original profile in Fig. 5a and the zero padded profile in Fig. 5b. This strategy is, however, suboptimal since the padded data is not representative of the actual profile, hence it significantly influences the roughness values obtained after parameter determination.

**Fig. 6:**
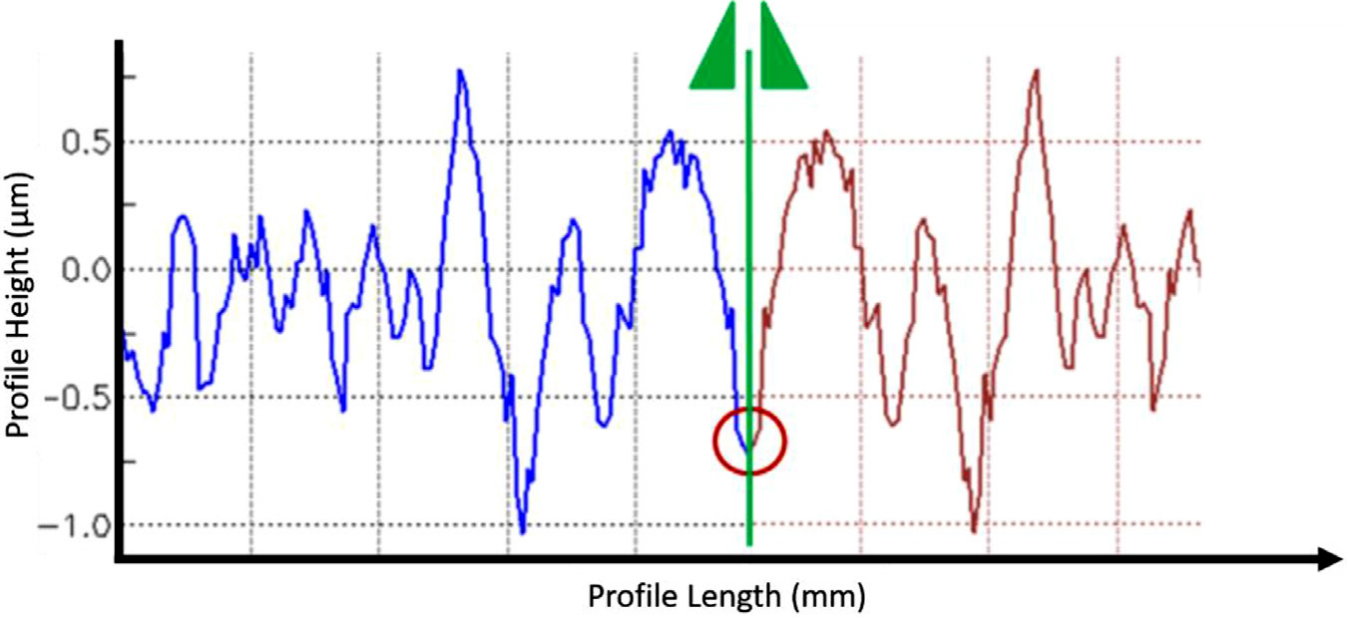
Close up of mirror padding - creates extra peaks/valleys.

“Mirror padding”, also described in ISO 16610–28:2016 [6], concerns mirroring a part of the profile across the endpoints. Whereas this is more representative, it also leads to an overemphasis of edge features (peaks, valley) since they are reproduced very close to one another [9]. Moreover, mirroring often inherently yields an extra peak or valley at the original endpoint, as can be seen on the left mirroring point of Fig. 5c that is shown enlarged in Fig. 6. Fig. 5d also shows that the resulting profile could be very different depending on the location where the mirroring occurs: compare Fig. 5c and 5d where the mirror points are shifted.

### Gaussian regression filter

**Fig. 7:**
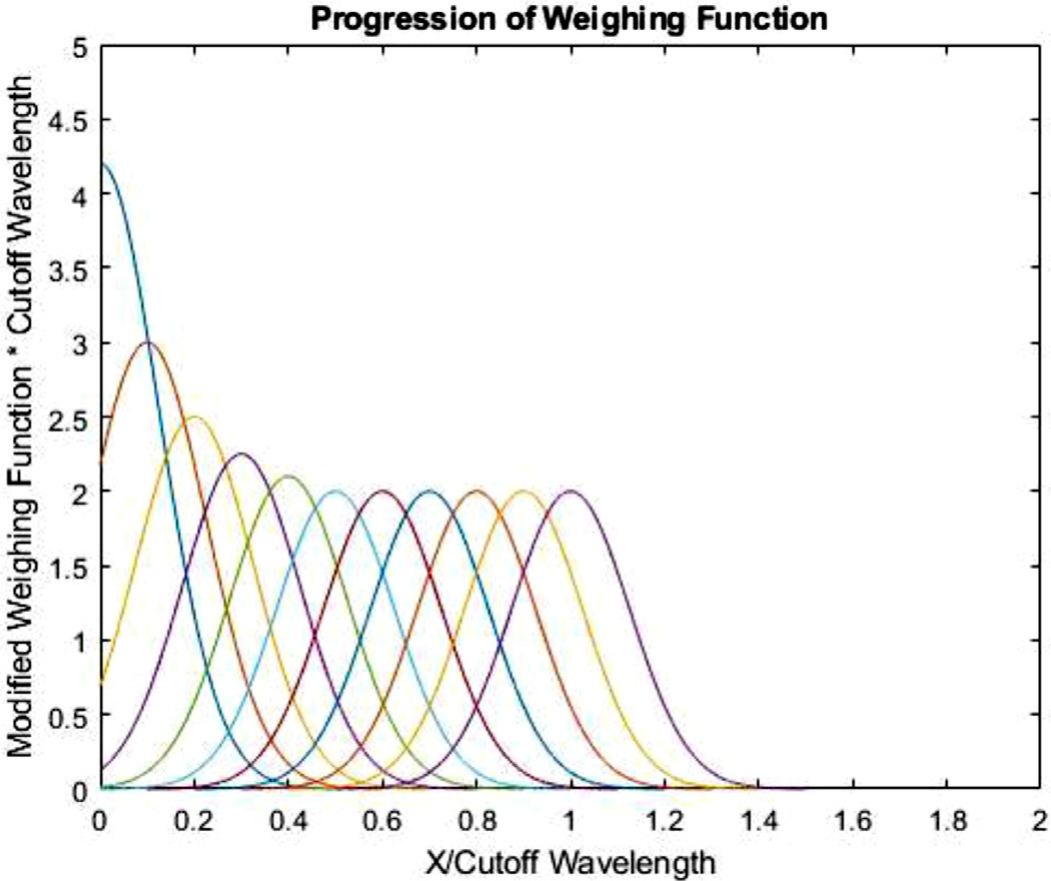
Progression of modified weighing function of a zero order Gaussian regression filter (after [10]).

It is to be noted that there are also a few newer filter types that inherently do not cause those “end effects”. The main one to be considered is the Gaussian Regression (GR) filter, where the weighting function is intrinsically modified in these “end effect” regions (one cutoff wavelength within either edge of the profile), in order to compute a value there (Fig. 7) [10]. Over the remainder of the profile the weighing function is unchanged. There are several versions of this filter, but most common are the zero and second order Gaussian Regression filters. The zero order GR filter is less computationally intensive, but deals with form less well than the more difficult to calculate second order GR filter. In absence of very apparent form, the zero order GR filter is more commonly used.

### Proposed method: padding of measurement tracks

#### General approach of padding

**Fig. 8:**
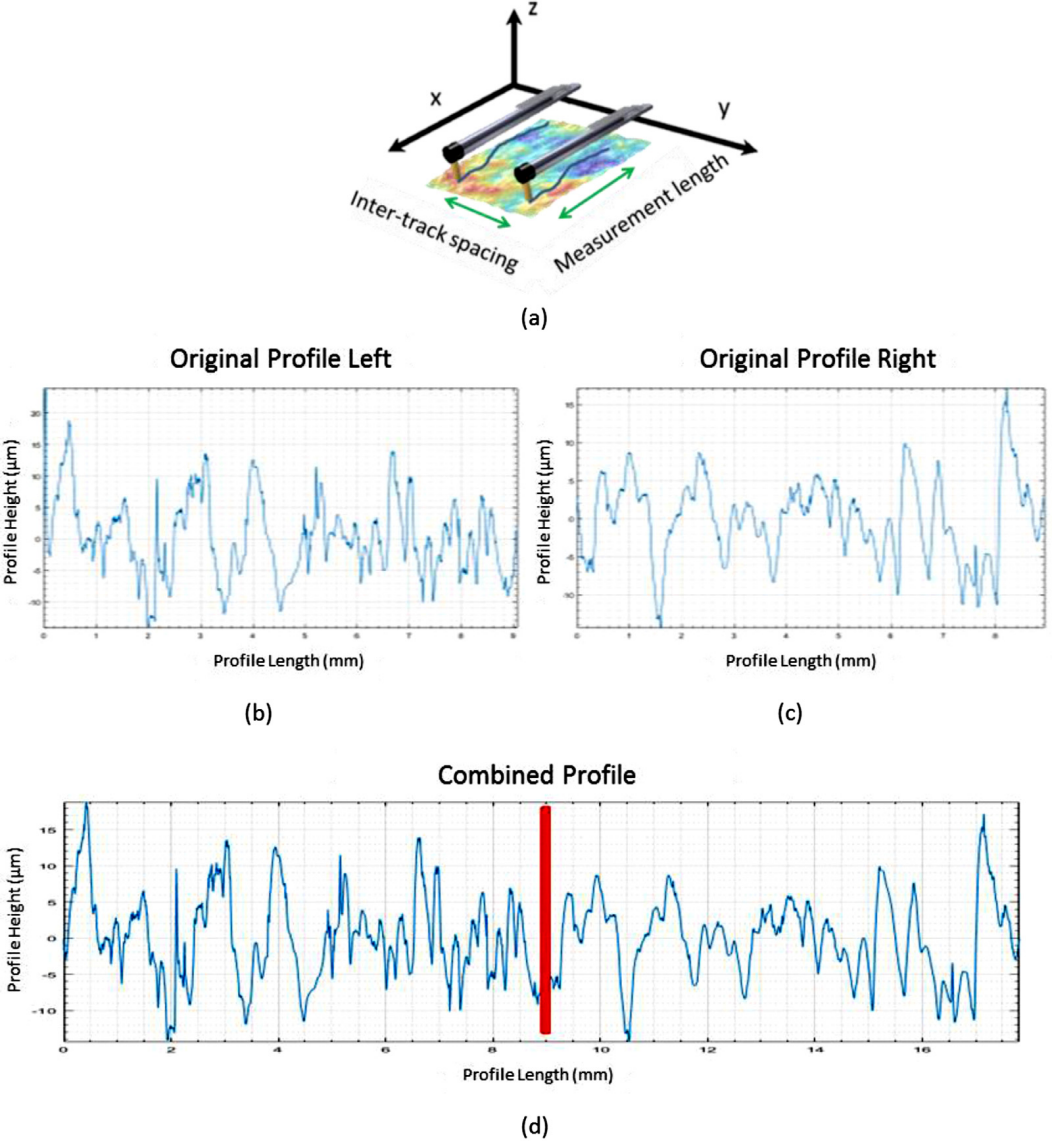
Parallel padding by measuring parallel profiles - see (a) (after [13]), (b) and (c) - and padding them into a pseudo-linear profile (d).

**Fig. 9:**
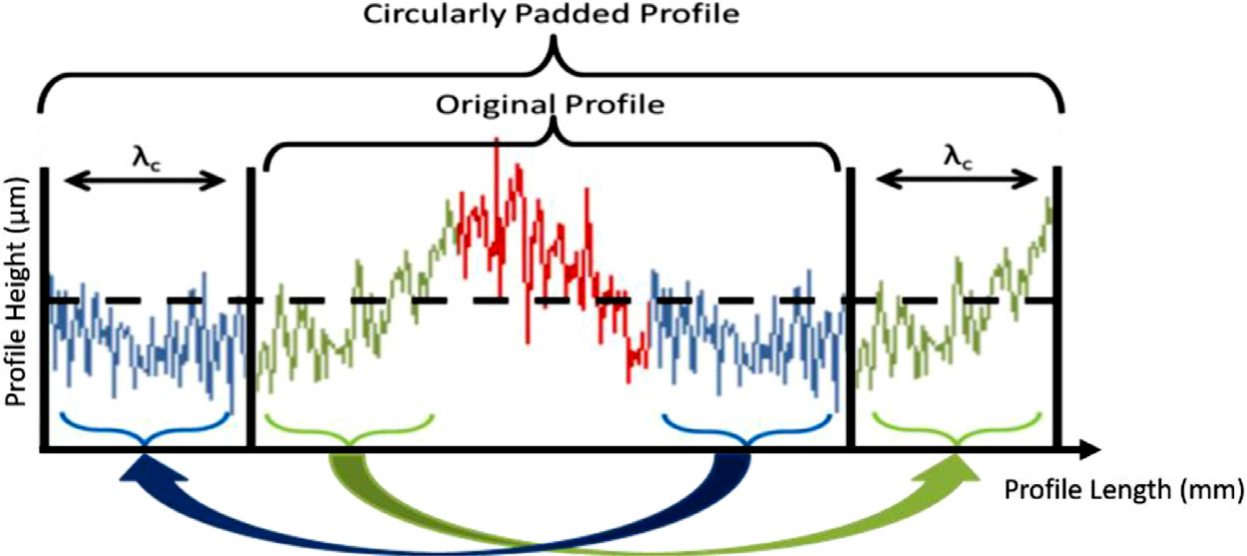
Circularly padded profile - treat the profile as a closed loop for the filtering step, after which the loop can be unwrapped again.

This paper discusses two padding strategies that avoid the mentioned biases. Unlike the GR filter, where the weighing function is tweaked to avoid “end effects”, these strategies are based on either appending actual data together or extrapolating representative data. If several short yet parallel tracks can be measured on the surface, they can be padded into a pseudo-linear profile using “parallel padding” (Fig. 8). This is even recommended by some profilometer manufacturers [11]. However, if the available space does not allow parallel tracks, a single track can be padded to itself, start-to-end, by “circular padding” (Fig. 9). Subsequently, the “circularly padded” profile can be unwrapped in a procedure similar to the ISO 16610–21:2011 [5] method of measuring roughness along the circumference of a cylindrical object [12]. This procedure conserves at both start and end a copy of the profile sufficiently long to maintain a sufficient measured length after filtering, i.e. at least *λ_c_* on either side.

If the fact that the original and filtered profiles are not of the same length is unwanted, circular padding can also be used on long enough profiles (i.e. ISO conform length) to simply maintain the full length after filtration.

#### Enforcing profile continuity

**Fig. 10:**
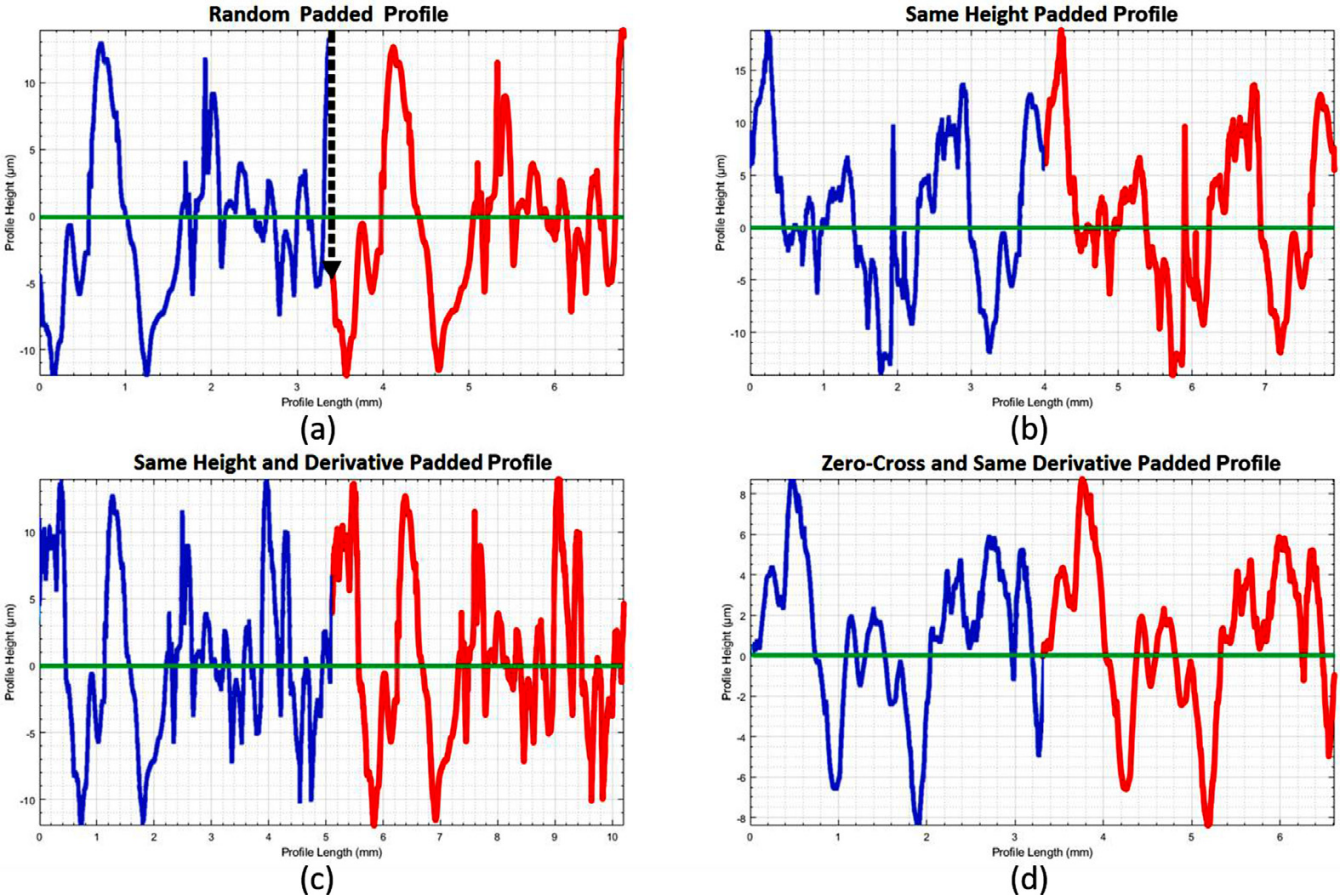
Padded profile continuity: random height and derivative (a), same height with different derivative sign (b), same height with same derivative sign (c), zero-crossing padding with same derivative sign (d).

It must be noted that for parallel or circular padding, unlike for the mirror-padding method, the junction between measured data and padding is not necessarily continuous, often resulting in jumps between data points (black arrow in Fig. 10a). It can be noted that a frequency domain based filtration (e.g. using Fourier) implies this situation, where the profile would repeat and discontinuities would therefore be present. These discontinuities may lead to artefacts in the profile that influence the roughness values, hence continuity has to be enforced. This can be achieved by trimming the padded profile to cover a track between profile points at the same height (Fig. 10b). To avoid an artificial peak or valley at the junction, equal signs of the trimmed profile derivative at the start and endpoint must moreover be enforced (Fig. 10c) [9]. Finally, to avoid influencing parameters that are highly sensitive to deviations from the zero-line (such as skewness, kurtosis, etc.), it is best practice to make this junction height a zero-crossing point (Fig. 10d). Only in cases where the padding length becomes too short a height different from zero should be used.

#### Filtering of form deviation

**Fig. 11:**
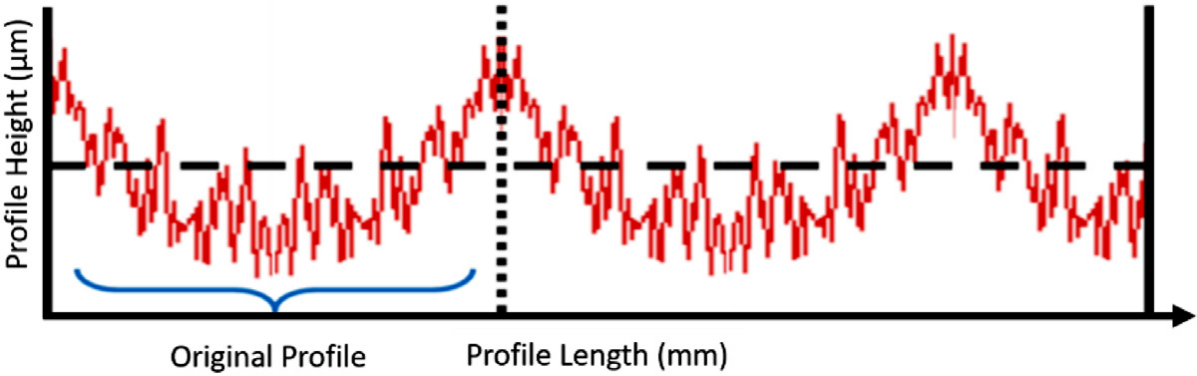
Padding of form features introduces a pattern that is not present in the actual part and thus not representative.

The primary profile can also comprise residual shape and form deviation information, if this was not adequately removed. If not removed before padding, a recurring pattern is introduced as an artefact (exemplified in Fig. 11). Therefore, is paramount that the form is corrected by applying a-priori knowledge (through the design such as a CAD model of the measured part) or by applying an appropriate filter. In cases where padding does introduce such a pattern, it will influence the waviness and roughness parameters. The extent of this influence on the resulting parameters depends on how close the padded length is to the cutoff wavelength used in the subsequent filtration to separate roughness and waviness.

#### Development of data post-processing and analysis tool

All of the above described adaptations to the normal ISO prescribed procedures were implemented in a Matlab software tool enabling the flexible and open manipulation and post-processing of linear profiles. This software tool enables the user to have full control of how the input profiles are treated, which cutoff wavelengths are used, which sections to ignore, etc. It also allows the user to append, pad, crop, filter, and analyze batches of measurement data. It also has the benefit of freedom for the parameters with which to filter, as sometimes the ISO prescribed cutoff wavelengths, which are often hard settings in measurement devices, might not be ideally suited.

## Validation approach and setup

This section will discuss the validation tests that were performed to verify whether results obtained from this padding method are significantly different from profiles filtered with standard methods. The method can be validated by verifying whether or not data processed by the circular padding method can be considered coherent with data processed using the ISO guidelines.

### Validation approach

Datasets are considered to be coherent with one another when their variances and their means do not significantly differ from one another. Equivalence of variance can be tested using a Fischer test, while equivalence of means can be tested by a Student *t*-test [14].

The validation of the parallel padding method is trivial. The data will always be representative of the surface, as it is not artificial. Instead of being in one line, it is a collection of tracks measured next to each other. This is not ideal, but definitely able to characterize the surface adequately, as long as specific known textures and features are filtered out, such as form, and proper continuity measures are taken at the transition points.

**Fig. 12:**
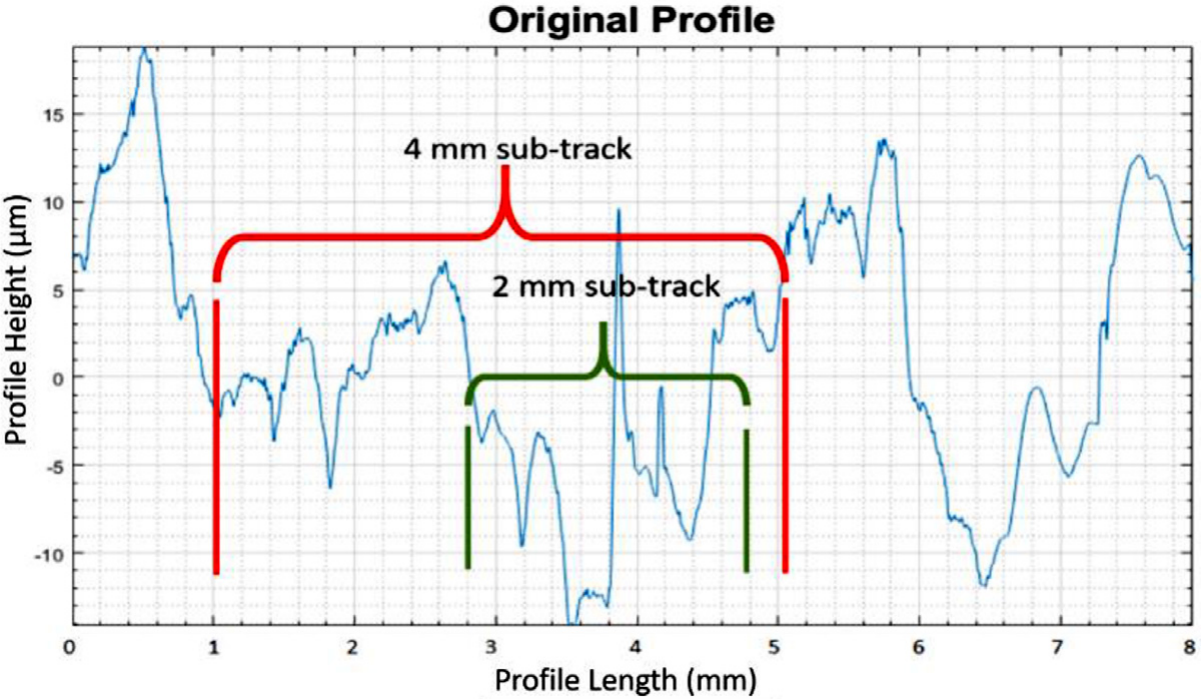
Example of the random sub-track selection on a 8 mm long profile.

The coherence tests (Fischer and Student) can be used to validate the circular padding method as follows. The idea is to obtain reference roughness values (according to the ISO standard) from measurements of sufficiently long tracks of a sample. Short subsections of these longer tracks with specifically fixed lengths are then randomly taken. These shorter tracks are analyzed using the circular padding (CP) method. Subsequent sets of these "sub-tracks" are gathered until the length of the sub-tracks is less than the appropriate cutoff wavelength, as shown in Fig. 12. The goal is to determine whether each collection of sub-tracks analyzed by CP is coherent and therefore consistent with the original not-padded (NP) collection of longer source tracks, with a reference ISO roughness. If coherent, this makes them indistinguishable from their longer counterparts and validates the CP method.

### Setup of the experiments

**Fig. 13:**
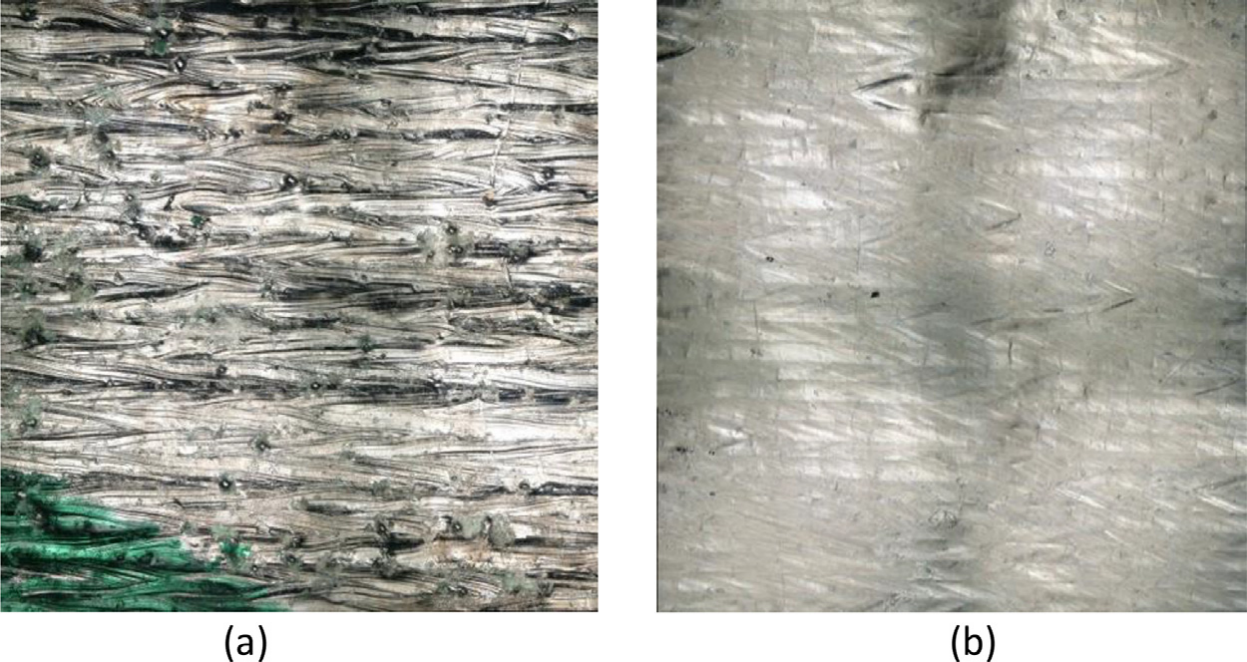
Optical Microscopy image of 3 mm by 3 mm areas of SLM-AM samples used for the measurements - Rough (a) and Smooth (b).

Measurements are taken on two additively-manufactured (AM) blocks, a rough one and a smooth one (Fig. 13). These AM blocks were produced by so called “powder bed fusion” (PBF) of 316 L Stainless Steel powder material, a process also referred to as selective laser melting (SLM) [15]. The smooth samples were obtained by laser remelting of the top layer (surface) of the SLM samples. The long profile tracks are measured parallel to one another and each have the same length (16 mm). The measurement tracks are taken at an angle of 45° to the molten tracks of the SLM process, in order to capture all relevant influencing factors on the surface roughness. The inter-track spacing between measured tracks is 200 µm. In total, 40 measured tracks are collected on each block.

**Graph 1:**
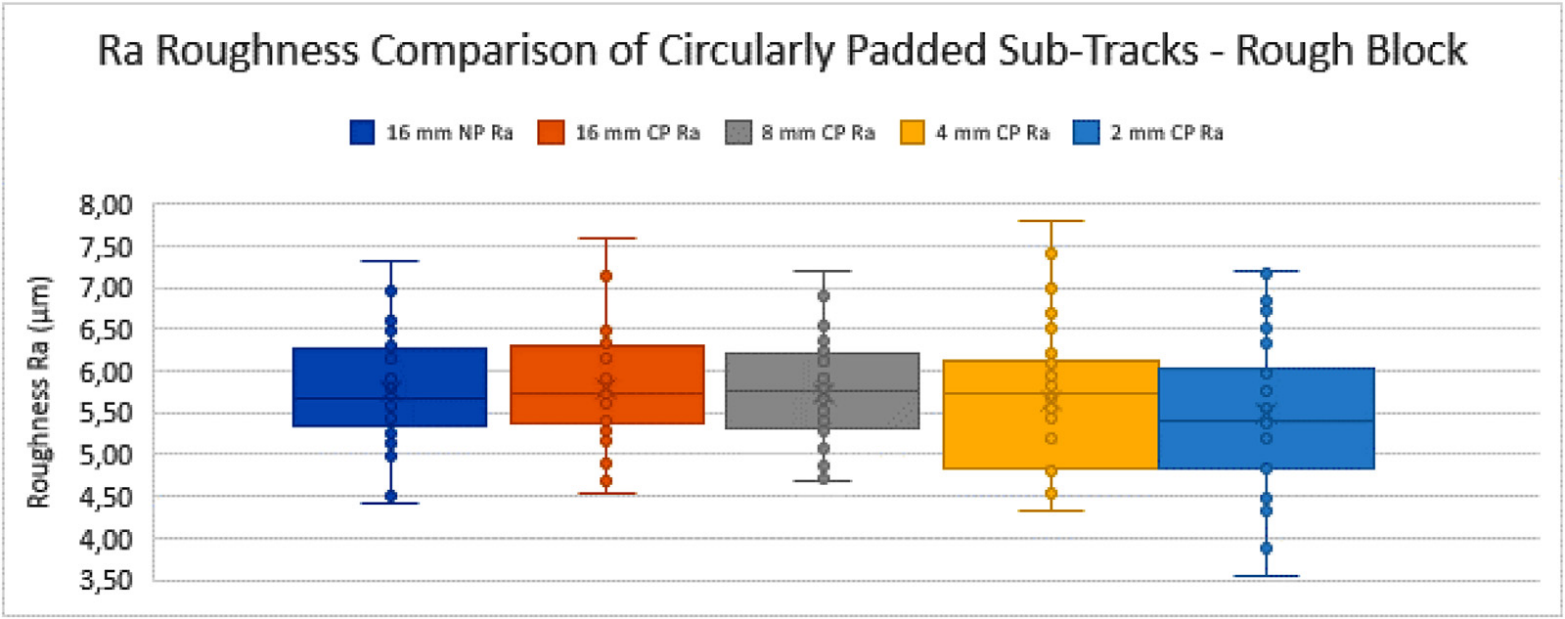
Ra Roughness Comparison of Circularly Padded Sub-Tracks - Rough Block.

**Graph 2:**
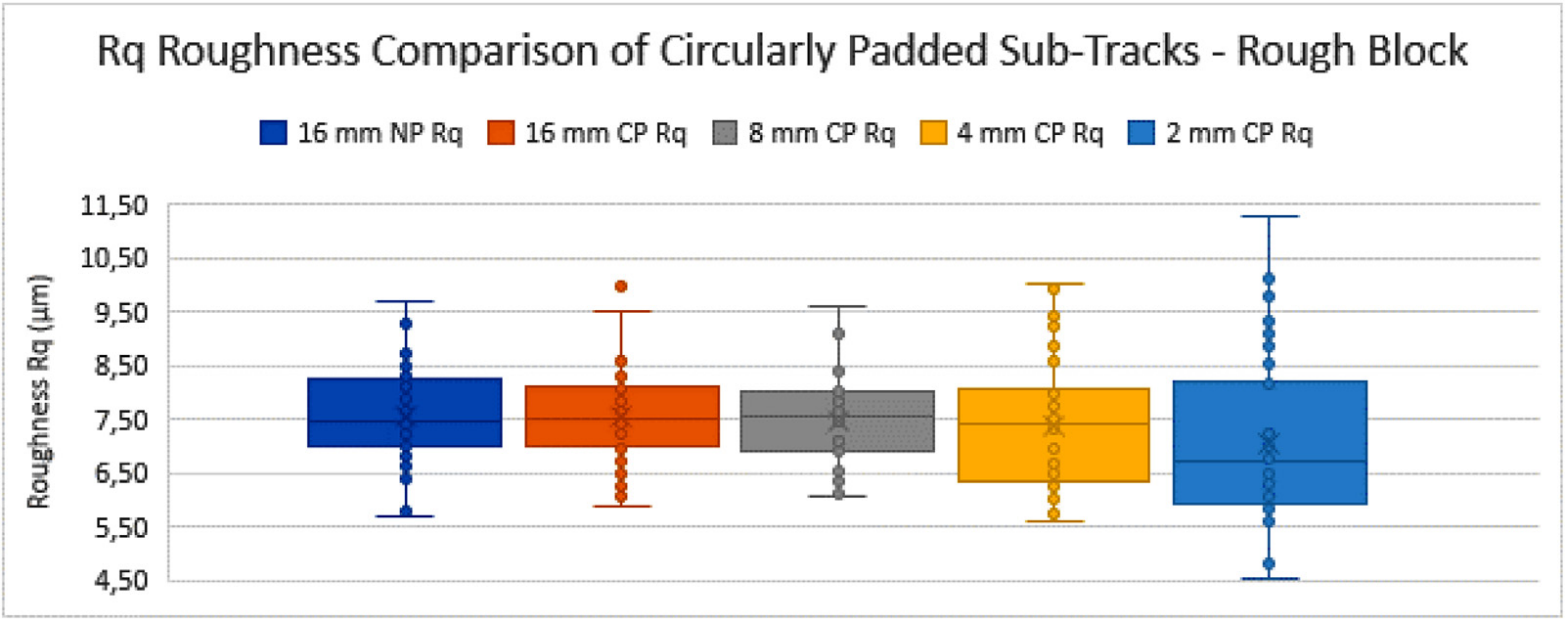
Rq Roughness Comparison of Circularly Padded Sub-Tracks - Rough Block.

The data is measured using a tactile 60° cone-shaped diamond stylus with a tip radius of 2 µm, mounted on a Taylor Hobson Talysurf profilometer device. The surfaces of the measured SLM-AM blocks show no distinctive manufacturing errors such as deep grooves or ridges. The *Ra* values of these blocks are larger than 2 µm and lower than 10 µm, thus according to ISO 4288 guidelines a cutoff wavelength *λ_c_* of 2,5 mm should be used. To acquire enough measurement data, the standard prescribes at least 12,5 mm (5 · *λ_c_*) measured track length (before filtration) [4] [7]. The actual initial measured track length is 16 mm. The subsequent sub-track lengths are halved every time, i.e. 8 mm, 4 mm and 2 mm.

All of these tracks are measured with identical settings and post-processed in an identical way within each dataset. For the NP tracks, no padding is used and the post-processing of the profile can be initiated immediately.

For the CP tracks, circular padding with zero-cross and preservation of the derivative sign is used. As mentioned before, this method pads the “end effect” regions as if the “zero-crossed/preserved-derivative” profile is looped. The initial profile length is then lengthened by at most 2 · *λ_c_*, one *λ_c_* on each end, to preserve the full length after filtration. The coherence tests are performed on both the *Ra* and *Rq* parameters.

## Results and discussion of validation tests

This section deals with the comparison of various techniques for dealing with profile parameter determination of shorter measurement tracks. Firstly, it compares the circularly padded (CP) profile parameter determination to the non-padded (NP) profile parameter determination. Secondly, it compares the outcome of Gaussian regression (GR) filters to those same NP results. Lastly, it contrasts the results of CP to the results of GR filtering.

### Comparison of non-padded and circularly-padded profiles

The NP profiles of 16 mm length are used as the initial starting tracks (16 mm-NP). Their roughness values are calculated as described above, according to the ISO standards. After this, they are circularly padded (16 mm-CP), to assess whether this operation of CP in itself significantly alters the measurement set mean and variance for profiles that are long enough. The other sets of sub-tracks (i.e. those of 8 mm, 4 mm and 2 mm track length) are also each circularly padded and compared to the initial set of 16 mm NP tracks. The results of this comparison can be seen in the tables and graphs below (Table 1, Graph 1, Table 2 and Graph 2).

**Table 1:** Table depicting the comparison and coherence tests of the *Ra* values gathered from sub-tracks after circular padding – rough block.

**Table 2:** Table depicting the comparison and coherence tests of the *Rq* values gathered from sub-tracks after circular padding – rough block.

**Graph 3:**
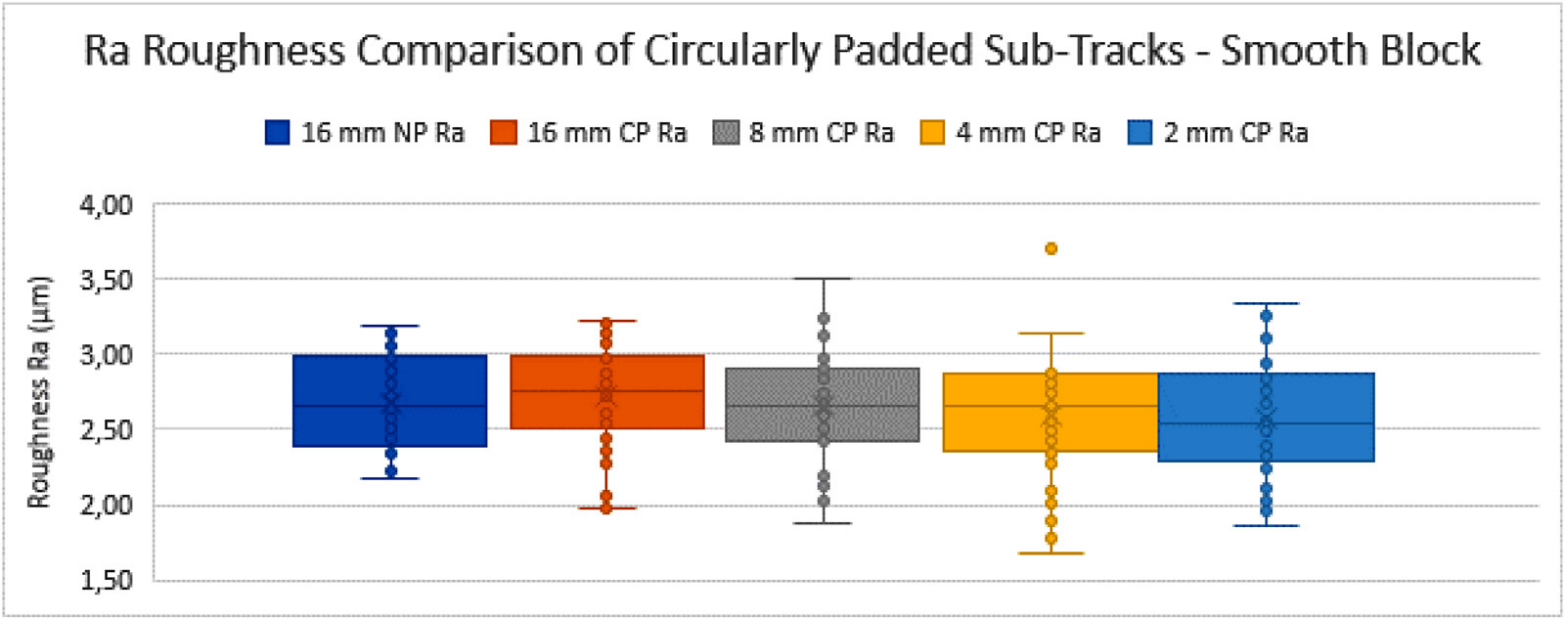
Ra Roughness Comparison of Circularly Padded Sub-Tracks - Smooth Block.

**Graph 4:**
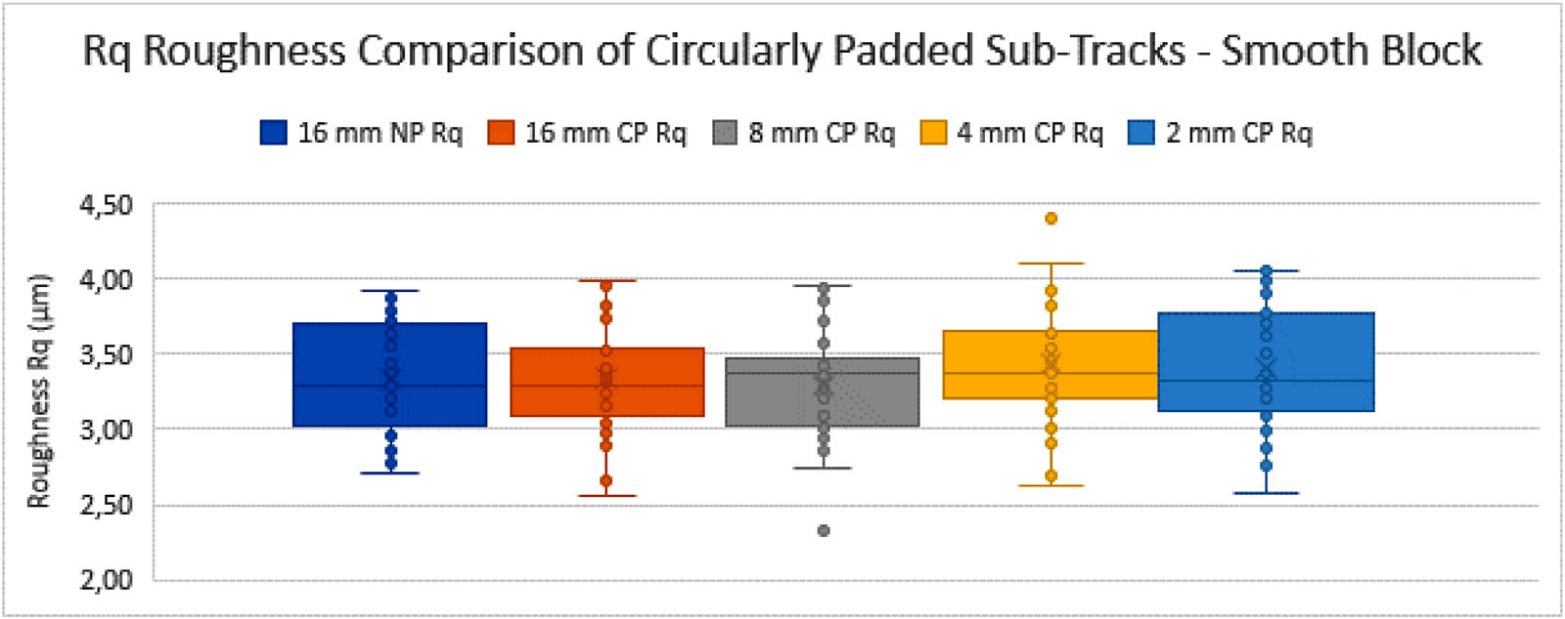
Rq Roughness Comparison of Circularly Padded Sub-Tracks - Smooth Block.

The Fischer test is performed to test the equality of variances of two measurement sets. For these sets, the sample sizes are the same, *n* = 40, and the degrees of freedom *f_1_* and *f_2_* of the two sets being compared are therefore 39. For a confidence level of 95% to declare incoherence, the ratio of the higher variance over the lower variance - the dimensionless *F*-value - should be higher than 1,7078 [16]. That would indicate a significant difference between the variances of the two compared data sets. If this is not the case, the hypothesis of incoherence of the variances is not accepted.

The *t*-test is then performed to test the equality of the means. Since the equality of the means is being tested, a two sided test needs to be used for the alternative hypothesis. Also, if the Fischer test above did not declare incoherence, a pooled estimate of the real variance can be used [14,17]. The degrees of freedom *f* of this pooled variance is 78. This means that for a confidence level of 95% to declare incoherence, for a two sided *t-*test, the dimensionless *T-*value (i.e. deviation of the means as compared to the standard deviation) should be higher than 1991 [18]. If this is not the case, the hypothesis of incoherence of the means is not accepted.

From these tables and graphs the following remarks and conclusions can be made for the “rough block”:

• All track measurement sets are compared to the 16 mm NP track measurement set for coherence.
• The trends are similar for both *Ra* and *Rq* parameters.
• The standard deviations tend to increase as the sub-tracks become shorter. This can be expected as the rough surface has high amplitude fluctuations in the surface at a relatively high wavelength (i.e. molten tracks from the manufacturing method, separated by approximately 120 µm measured at 45°, resulting in a distance of approximately 170 µm). Due to the random selection of the sub-track, a specific very high or low feature such as an edge between molten tracks (so called “weld lines”) might (not) be included, causing the standard deviation of the measurement set to increase.
• Both the 4 mm CP and 2 mm CP track measurement sets are shown to be incoherent with the 16 mm NP track measurement set in terms of their variance with 95% confidence (Fischer-test). This is again due to the standard deviations increasing as the tracks become shorter, as explained above.
• The comparison of the means (*t*-test) did not lead to a conclusion of incoherence of the means of any measurement set compared to the 16 mm NP track measurement set.
• This means that for this sample (the rough SLM block), as a preliminary conclusion, the CP method can only be used reliably to measure down to a track length of 8 mm, where according to the standard a track length of 12,5 mm is required. This limited improvement is due to the high amplitude of the features present in the measurement tracks, which increase the variance of the measurements too much to make a reliable conclusion if the measured track length is shortened drastically.

The same analysis was done for the “smooth block”, which has a lower roughness value, but still falls within same filter settings (*λ_c_*) category according to ISO 4288, as the *Ra* value has a mean of 2,67 µm. The results are given in Table 3, Graph 3, Table 4 and Graph 4. Notice that for this smooth sample the test never concluded to incoherence in variance nor to incoherence in means.

**Table 3:** Table depicting the comparison and coherence tests of the *Ra* values gathered from sub-tracks after circular padding - smooth block.

**Table 4:** Table depicting the comparison and coherence tests of the *Rq* values gathered from sub-tracks after circular padding - smooth block.

The following remarks and conclusions are drafted from the results regarding the “smooth block”:

• All track measurement sets are again compared to the 16 mm NP track measurement set for coherence.
• The trends are again similar for both *Ra* and *Rq* parameters.
• The standard deviations tend to become slightly bigger as the sub-tracks become shorter. The effect where a shorter measurement track yields a larger standard deviation due to specific features (not) being included is also much less apparent here, because the surface has a much lower roughness value (both *Ra* and Rq) and less high amplitude-high wavelength features such as weld line edges.
• The comparison of the variances (Fischer-test) did not lead to a conclusion of incoherence of variances of any measurement set compared to the 16 mm NP track measurement set, with 95% confidence.
• The comparison of the means (*t*-test) did not lead to a conclusion of incoherence of the means of any measurement set compared to the 16 mm NP track measurement set, with 95% confidence.
• This means that, for the smooth block tested here, the tracks of 2 mm measured length which are circularly padded can be considered coherent with the tracks of 16 mm measured length. Therefore, the CP method can be used to reliably assess short samples that are expected to have similar roughness profiles, due to being made with similar parameters in a similar process.

The standard deviations for the smooth sample are noticeably lower than those for the rough sample. This is the reason that the Fisher-tests for the smooth sample do not conclude incoherence with 95% confidence. A summary of all standard deviations is given in Table 5.

**Table 5:** Standard deviations summary for the rough and smooth samples.

Standard Deviations Summary				
Series	*Ra* (µm)		*Rq* (µm)	
	Rough	Smooth	Rough	Smooth
16 mm NP	0,618	0,321	0,862	0,379
16 mm CP	0,628	0,347	0,855	0,333
8 mm CP	0,612	0,383	0,902	0,344
4 mm CP	0,855	0,392	1196	0,373
2 mm CP	0,955	0,378	1580	0,397

These tests reinforce the notion that it should always be tested beforehand whether the circular padding method is suitable for the typical surface topography at hand. This can be done through coherence testing, as was shown here.

### Comparison of non-padded and Gaussian regression filtered profiles

The comparison with the above mentioned zero order Gaussian Regression (GR) filter can also be made. The same approach is considered, where the same sub-tracks are filtered according to the GR method, using the same *λ_c_* as before (2,5 mm). Coherence tests comparing the results to the original 16 mm measurement track set are then performed. The test is performed on exactly the same sub-tracks as those used for the CP method. The results are given in Tables 6 and 7 for the rough AM block, and in Tables 8 and 9 for the smooth AM block.

**Table 6:** Comparison and coherence tests of the *Ra* values gathered from sub-tracks after zero order GR filter – rough block.

**Table 7:** Comparison and coherence tests of the *Rq* values gathered from sub-tracks after zero order GR filter – rough block.

**Table 8:** Comparison and coherence tests of the *Ra* values gathered from sub-tracks after zero order GR filter – smooth block.

**Table 9:** Comparison and coherence tests of the *Rq* values gathered from sub-tracks after zero order GR filter – smooth block.

With respect to the comparison between the circularly padded results and the zero order GR results, the following remarks can be made:

• Again all track measurement sets are compared to the 16 mm NP track measurement set for coherence.
• The standard deviation tends to increase as the sub-tracks become shorter. This effect is again less pronounced with the smooth sample due to lower amplitudes and shorter wavelengths of features in the smooth block measurement tracks.
• Both the 4 mm GR and 2 mm GR track measurement sets are shown to be incoherent with the 16 mm NP track measurement set in terms of variance with 95% confidence (Fischer-test) in the rough block. This is in good agreement with the results from the CP method described above.
• For the smooth block, the 2 mm GR track measurement set for *Ra* is shown to be incoherent with the 16 mm NP track measurement set with 95% confidence in terms of variance. The fact that this measurement set is incoherent whereas the CP measurement set was not, can be attributed to the GR method altering the weighing function in the first and last *λ_c_* of the profile. It is in these “end effect” regions of the profile where the GR method will give different results when compared to the CP method. The shorter the measurement track, the larger the ratio of “end effect” region to “normally filtered” region, resulting in the CP and GR results possibly deviating more from one another.

### Comparison of circularly padded and Gaussian regression filtered profiles

The CP and GR filter methods can also be directly compared for coherence to check their degree of agreement in more detail. The result of this analysis is given in Table 10 for the *Ra* values and Table 11 for the *Rq* values.

**Table 10:** Comparison and coherence tests of the *Ra* values gathered from sub-tracks with CP and GR methods.

**Table 11:** Comparison and coherence tests of the *Rq* values gathered from sub-tracks with CP and GR methods.

In this analysis the calculated *Ra* and *Rq* roughness values for the same track lengths are compared between both the CP and GR methods. The two methods are found to be very similar, with only the 8 mm measurement length set indicating incoherence of the means with 95% confidence. It can be concluded that the two methods give consistent results. As the GR method is often considered as an adequate solution for “end effects”, this study demonstrates that the CP method can provide a good alternative as well.

The choice of which filter to use depends highly on how the user wants to treat the profiles. The Gaussian Regression filter uses a modified weighing function to deal with the “end effect” regions, which is more difficult to interpret. The circular padding method tries to predict a set of padding data before and after the measured profile, as representative of the actual surface as possible, essentially “looping” the profile around. It maintains the same filtration characteristics throughout. If the goal of the assessment is to treat the end effects with a similar weight as the rest of the profiles, CP would be preferential.

## Conclusions and best practices

**Fig. 14:**
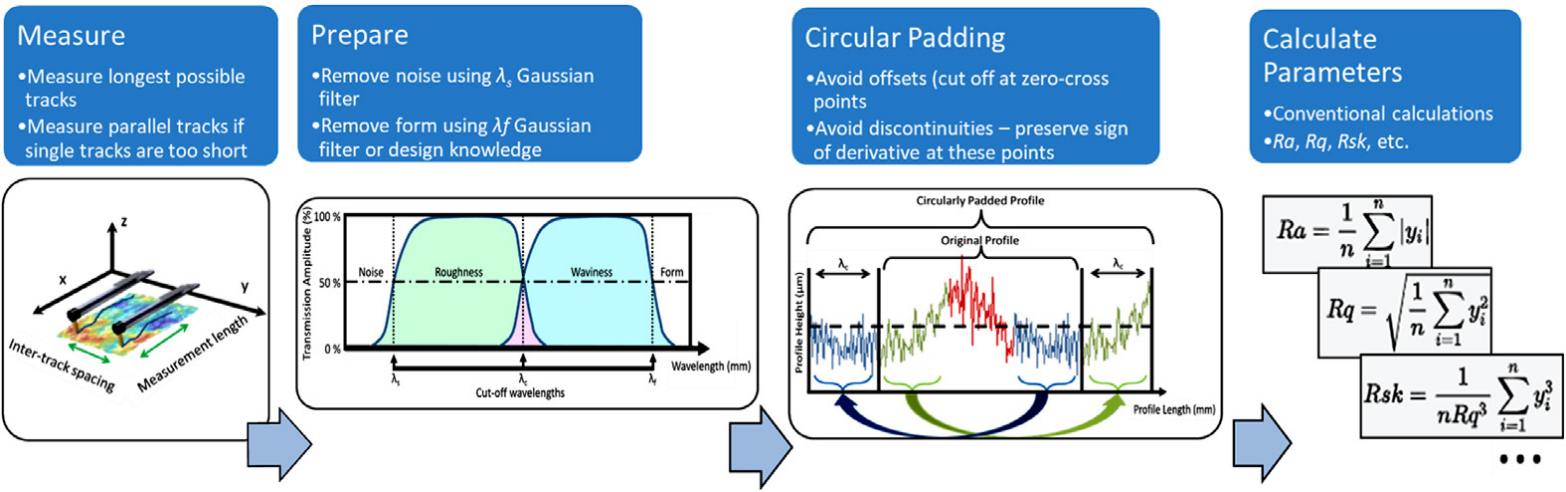
Flowchart depicting the workflow from measurement to calculated parameters using the circular padding method described and assessed in this work.

In this paper, a method is proposed for using a circular padding strategy to measure small track length roughness values, where rigid application of the ISO standards appears not appropriate. It is also explained why, when using the circular padding method, care has to be taken with regards to the junction points between appended or padded profiles, to not introduce unwanted effects and to get a profile that is as representative of the actual surface as possible. A flow chart of the method is provided in Fig. 14.

Validation tests on rough and smooth additively manufactured samples demonstrated that roughness measurements on circularly padded tracks are in good agreement with ISO standardized roughness measurements.

It is also shown that the circular padding method agrees well with and can be used as an alternative for the Gaussian Regression method, which is mentioned in the standards. The circular padding method is shown to be very useful in correctly assessing parts with short measurement lengths, such as those often found in additively manufactured parts. Component miniaturization is a current trend, and as such methods that can measure roughness on increasingly smaller samples become very valuable in the quality optimization process.

The method should always be validated on a representative set of data before application on similar samples, if available, as this is shown to be vital to use the method properly. The validation identifies how short measurement tracks can be considered to still be in agreement with ISO standard procedures. It is recommended to always use a separate object, with similar surface but long enough according to the ISO standard procedures, to assess the usefulness of CP. If the difference between the results obtained with CP and another accepted default method is not deemed significant, it can be assumed CP will give comparable and acceptable results. In future work it can be investigated whether or not curvature radius and other morphological aspects are seriously affected - or even compromised - by using this method.

**References:** *ISO 16610: Geometrical product specifications (GPS) - Filtration - Part 28: Profile filters: End effects*

## Declaration of Competing Interest

The authors declare that they have no known competing financial interests or personal relationships that could have appeared to influence the work reported in this paper.
